# Higuchi Fractal Dimension as a Method for Assessing Response to Sound Stimuli in Patients with Diffuse Axonal Brain Injury

**DOI:** 10.17691/stm2020.12.4.08

**Published:** 2020-08-27

**Authors:** K.V. Gladun

**Affiliations:** Junior Researcher, Human Higher Nervous Activity Laboratory Institute of Higher Nervous Activity and Neurophysiology, Russian Academy of Sciences, 5A Butlerova St., Moscow, 117485, Russia

**Keywords:** Higuchi fractal dimension, diffuse axonal brain injury, electroencephalography.

## Abstract

**The aim of the research** was to study the fractal dimension of the EEG signal by the Higuchi’s method in patients with diffuse axonal injury (DAI) of the brain.

**Materials and Methods.:**

The study was performed in 28 patients with DAI of different severity and 13 sex- and age-matched controls. The Higuchi’s method of fractal dimension was used to investigate brain response to sound stimuli of different emotional coloring as well as the features of the EEG signal in the resting state.

**Results.:**

The EEG data demonstrated the highest values of fractal dimension in patients with DAI in the resting state. The values of fractal dimension in different emotional states considerably differ both in healthy subjects and in those with DAI. An increase in fractal dimension in response to stimuli occurs predominantly at the frequency of the theta rhythm in the control group and the frequency of the alpha rhythm in the patients with severe DAI.

**Conclusion.:**

Higuchi fractal dimension can be used as a complementary diagnostic tool that allows differentiating perception of emotionally significant audio information in patients with brain injury.

## Introduction

Many biological systems have complex nonlinear features and cannot be investigated in full only with the use of the theory of linear systems. The works dealing with the analysis of EEG signals from the point of view of the theory of dynamic systems, particularly, deterministic chaos, allow a new approach to the understanding of neurophysiological processes [1–4]. The deterministic chaos of the brain bioelectrical activity is manifested by the non-periodicity and complexity of the EEG pattern under various conditions.

Electroencephalography allows recording a pooled signal of many neurons with specific phase and spatial dynamics. EEG signals vary even in response to simple sound stimuli and, at first sight, have a random character. However, they do not. Variability of the stimuli depends on the individual features of the higher human nervous activity. An individual demonstrates a sustainable and reproducible response to the same kind of a stimulus. Similar sustainability of individual response with interindividual variability is inherent in the nervous system at all its levels [1, 2].

EEG properties include constant parameters inherent in a particular state of consciousness (for example, wakefulness or sleep) as well as dynamic indicators: a change in the amplitude of rhythms under various cognitive load or a change in the dominant frequency in particular areas of the cerebral cortex depending on a sleep or wakefulness phase which determine a variety of approaches to signal processing. Thus, for example, neuron activity in the nervous system of the shellfish is explained in terms of the chaos theory [3]. Activities of single neurons [4] and groups of neurons [5], as well as the work of ion channels [6] in mammals, are described with the methods of nonlinear dynamics.

Higuchi’s method for calculating fractal dimension (HFD) may be used as a nonlinear measurement for the analysis of biological signals [7]. HFD method estimates the dimension of D fractal (or Hausdorff space) of the basic time set where the given dimension measures a degree of “roughness” of the fractal form and varies in the range of values from 1 to 2. The advantage of calculating fractal dimension by Higuchi’s method was found to be preferable compared to traditional methods based on the EEG-signal power spectrum or its versions. In spite of no difference in the results obtained by the Higuchi’s method and the box counting-method [1], faster data pre-processing and less computing power used make it possible to opt for the Higuchi’s method.

The human EEG study in the state of wakefulness and sleep by the HFD method demonstrated its effectiveness on long signal segments [8, 9]. The studies of human and animal EEG with injury to the brain by the HFD method are few in number compared to a large number of studies where other nonlinear methods are used [10–12]. In the study by Spasić (2005–2010) [13, 14] the HFD method was first applied to calculate fractal dimension of EEG signal in rats with injury to the brain. Kekovic et al. [15] described a mechanism of the development of Alzheimer’s disease having established a diffuse neurotoxic effect of chemicals on rats’ brains by the HFD method of calculating.

The issues considered in our article are related to the identification of nonlinear dynamic properties of the brain based on the quantitative EEG data.

**The aim of this study** was to investigate brain activity on the base of the dynamics of the Higuchi fractal dimension in response to sound stimuli and at the resting state in patients with diffuse axonal injury (DAI) and healthy volunteers.

We believe that misidentification of a specific type of emotion (positive and negative) on the background of low general emotional state can affect the course of a traumatic illness.

## Materials and Methods

### Study participants.

This work was supported by the Institute of Higher Nervous Activity and Neurophysiology of the Russian Academy of Sciences on the basis of the Central Clinical Hospital of the Russian Academy of Sciences and the Salus-Polus Medical Center (Moscow) from 2013 to 2019.

The criteria for participation of the patients were: males aged 20–55 diagnosed with “diffuse axonal injury to the brain in a mild and severe condition”. The biomechanics of impact trauma included abrupt acceleration and braking of the head in a road traffic accident. The diagnosis of DAI was established on the base of comprehensive assessment of the neurovisualization data and neurological status.

The choice of the patients was determined by a few factors. DAI allows studying a comprehensive brain response excluding the effect of the parameters that are difficult to control and connected with localization, volume, a degree of compensatory processes in the focal and contralateral areas which undoubtedly have an impact on the bioelectrical response of the cerebral cortex. Moreover, a change in the human brain activity because of DAI due to a traumatic brain injury has similar features with Alzheimer’s disease that worsen a risk of its occurrence [16, 17]. That increases interest in the research in this field.

The criteria for the involvement of healthy subjects were: males aged 20–55. All the participants of the control group were examined for the presence of psychological/psychiatric disorders, none of them reported drug or alcohol use.

Exclusion criteria (for the patients and healthy subjects): history of neurological and psychiatric diseases, post-traumatic epilepsy, focal lesion or necrosis, hearing impairment, taking medications that affect the emotional background. All the participants underwent an independent double examination with epileptologist.

All the patients underwent MRI or CT on admission within 2 to 10 h after traumatic exposure and again after 5 to 10 days. A 1.5 T MRI system was used.

The sequences of MRI included T1 and T2 weighted values, fluid attenuation inversion recovery (FLAIR) mode, and diffusion-weighted imaging (DWI) to verify the diagnosis of DAI. CT examination of the brain was performed on the Somatom CR and Somatom AR tomographs (Siemens, Germany) with a 5 mm step.

The MRI and CT data were reviewed independently with two radiologists. The patients’ condition on admission was assessed at 12–14 points on the Glasgow Coma Scale (GCS) for mild DAI and 6–8 points for severe DAI. At the time of EEG recording, the patients’ condition was assessed at 13–15 points for mild DAI and 7–12 points for severe DAI.

Three groups were formed: the 1^st^ control group (n=13; average age — 31.4 years); the 2^nd^ group with mild DAI (n=13; average age — 31.6 years; 13–15 points on the GCS); the 3^rd^ group with severe DAI (n=15; average age — 28.7 years; 7–12 points on the GCS). All the study participants had higher education. The subjects’ clinical and demographic profiles are presented in Table 1.

**Table 1:** Clinical and demographic profile of the study participants

Participants	Age	Group	GCS at admission (points)	GCS at taking EEG (points)	Neurovisualization (1^st^ examination)	Time of testing after suffering an injury (hours)	Neurovisualization (2^nd^ examination)	Time of testing after suffering an injury (days)
***Healthy volunteers (control)***								
1	20	1	—	—	—	—	—	—
2	25	1	—	—	—	—	—	—
3	55	1	—	—	—	—	—	—
4	20	1	—	—	—	—	—	—
5	23	1	—	—	—	—	—	—
6	20	1	—	—	—	—	—	—
7	28	1	—	—	—	—	—	—
8	25	1	—	—	—	—	—	—
9	29	1	—	—	—	—	—	—
10	31	1	—	—	—	—	—	—
11	47	1	—	—	—	—	—	—
12	45	1	—	—	—	—	—	—
13	40	1	—	—	—	—	—	—
***Patients with diffuse axonal injury***								
14	20	2	13	13	CT	2	MRI	7
15	31	2	12	13	CT	2	MRI	10
16	40	2	13	15	CT	3	MRI	7
17	29	2	13	13	CT	2	MRI	7
18	28	2	12	13	CT	2	MRI	5
19	30	2	13	15	CT	2	MRI	10
20	43	2	13	15	CT	5	MRI	5
21	26	2	13	13	MRI	10	MRI	6
22	27	2	14	15	CТ	3	MRI	7
23	25	2	13	12	CТ	2	MRI	7
24	40	2	12	13	MRI	9	MRI	7
25	36	2	13	13	CТ	3	MRI	7
26	32	2	12	15	CТ	8	MRI	10
27	31	3	6	9	MRI	2	MRI	8
28	20	3	9	12	MRI	6	MRI	7
29	26	3	8	10	MRI	2	MRI	7
30	28	3	8	10	MRI	2	MRI	10
31	24	3	7	7	MRI	2	MRI	9
32	20	3	6	8	MRI	2	MRI	9
33	20	3	8	11	MRI	2	MRI	8
34	22	3	6	8	MRI	2	MRI	7
35	40	3	7	7	MRI	2	MRI	5
36	40	3	8	11	MRI	3	MRI	9
37	34	3	8	10	MRI	2	MRI	8
38	24	3	8	10	MRI	4	MRI	10
39	43	3	9	12	CТ	2	MRI	8
40	24	3	9	12	MRI	2	MRI	6
41	35	3	6	7	MRI	2	MRI	7

### Ethical aspects.

The research plan was approved by the Ethics Committee of the Central Clinical Hospital of the Russian Academy of Sciences and the Ethics Committee of the Institute of Higher Nervous Activity and Neurophysiology of the Russian Academy of Sciences. All the participants (or their representatives) signed informed consent to participate in the study. The information related to medical secrecy and patient confidentiality underwent data depersonalization and code assignment procedures.

### Sound stimuli.

The sounds used in the study were taken from the International Affective Digitized Sounds database [18]. HFD was analyzed on presentation of the sounds of various emotional coloring. Six types of stimuli with the following symbols: “unpleasant noise”, “cough”, “laughter”, “crying”, “barking”, “bird singing” were used. Each type of a stimulus was presented in three variants. The control EEG epoch was recorded in a state of quiet wakefulness with closed eyes and lasted 90 s. The Presentation software package (Neurobehavioral Systems, Inc., USA) was used to present sounds in a random order; each type of stimulus lasted 10 s and was repeated 12 times. The study time ranged from 30 to 60 min (recording was suspended in case of visible signs of nervous exhaustion in the patients). According to the types of stimuli, the recording was divided, followed by subsequent connection into an epoch of 60 s for a specific type of a stimulus.

### EEG recording.

EEG was recorded from 19 channels located in accordance with the international 10–20 system, with unipolar montage using the Encephalan portable electroencephalograph (Medicom MTD, Russia). The electrode resistance was kept below 10 kΩ. The sampling rate was 250 Hz. EEG was recorded in the patients on days 3–4 after having a traumatic brain injury. Lateralization was assessed with preliminary exclusion of the medial channels and division of 16 channels into two parts corresponding to the right and left hemispheres of the brain. The 16 channels were divided into 8 pairs: Fp1–Fp2; F7–F8; F3–F4; T3–T4; C3–C4; T5–T6; P3–P4; O1–O2. The oculogram was recorded from two electrodes placed 1 cm above and below the left eye. The artifacts were removed with the EEGLAB toolbox [19].

### Fractal dimension.

A fractal curve is a curve that has the property of self-similarity, i.e., each of its fragments retains the same general pattern at the reduction of the scale of this curve. The EEG signal from the point of view of the object is not endowed with the property of a true fractal since it has properties of self-similarity not in all its scales. The HFD for the EEG signal was calculated according to the original Higuchi’s algorithm [7]. In the context of this article, the term HFD is a measure of the complexity of a curve. Thus, the higher the HFD value, the higher the signal complexity and, consequently, the lower its fractal properties [7]. The EEG signal for each subject is divided into 7 states: 6 of them are response to sound stimuli and 1 — to the resting state. To determine significant results in all the clinical frequency ranges, the following frequency ranges were filtered at calculating HFD: 2–20 Hz, slow-wave — 2–7 Hz, alpha rhythm — 8–13 Hz, beta rhythm — 14–19 Hz. The following conditions were observed at work: k_max_ parameter of the Higuchi’s algorithm was 8; filtering frequency range width was 5 Hz; EEG epoch duration was 60 s. The intergroup comparison of the mean HFD values for all the stimuli between the subjects was performed. Within each group, the mean HFD values for each stimulus were compared with each other and for each stimulus with the resting state. To exclude the influence of factors associated with nervous exhaustion and/or general fatigue, two consecutive periods of 30 s each were analyzed. The EEG segments corresponding to the first and second (30 s each) parts of the EEG study had the same HFD values for each compared state. Further results were calculated for the fragments of 60 s duration. Statistical equations were calculated using the SPSS v.  14.0 software. Based on the data obtained for the three independent groups, multivariate analysis of variance (MANOVA) was carried out for 21 independent variables in accordance with the factors: group, state in response to a stimulus, hemisphere, localization, and channel. The dataset underwent the Kolmogorov–Smirnov test and the data fit a normal distribution. Assumption was checked for the same variance for each group (category) of the independent variable by the Leuven test. The Leuven statistics turned out to be insignificant at p>0.05, which confirms the null hypothesis that the groups have equal variances. The statistical analysis of the differences in the HFD values was carried out between each of the seven states, depending on the cerebral hemisphere (Fp1–F7–F3–T3–C3–T5–P3–O1 — left hemisphere; Fp2–F8–F4–T4–C4–T6– P4–O2 — right hemisphere), the area corresponding to the eight localizations (Fp1–Fp2; F7–F8; F3–F4; T3–T4; C3–C4; T5–T6; P3–P4; O1–O2) and each of the sixteen channels (Fp1, ..., O2). Multivariate comparison tests allowed identifying independent variables significantly associated with the dependent variable (Tukey, Bonferroni, Dunnett). The data for each filtering band (2–20, 2–7, 8–13, 14–19 Hz) underwent the described protocol.

### Assessment of the effect of education level and age on HFD.

The group of fixed variables indicating the level of education included 3 values: 1 corresponded to 14 years of study; 2 — 15 years, 3 — 17 years, and more. Besides, three variables were taken to indicate the age of the participants, where the first variable included participants aged 20 to 31, the second — from 32 to 40 years, and the third — from 41 to 55 years. The GLM Univariate ANOVA analysis was performed for each dependent variable corresponding to each frequency. The regression analysis, analysis of deviations for each dependent variable for the factors “group”, “stimulus”, “age”, and “educational level” revealed significant differences for the variables in the following frequency ranges: 2–20, 2–7, and 8–13 Hz (the significance level of the general F-test p=(1–0.951)/18=0.003 for assessing differences between 6 stimuli in the three groups). Multivariate ANOVA values were assessed by Bonferroni correction test, significance level p=(1–0.951)/162=0.0003 (assessment of differences between 6 variables for each of the stimuli; 3 variables corresponding to the group factor; 3 variables reflecting age; 3 variables reflecting the level of education). The factors of group, age, and level of education had three independent subgroups. The two-sided Dunnett test was used to compare each of the two subgroups with the third one. The HFD values without sound stimulation varied significantly between different groups of participants (by age and education level), however, when analyzing the HFD in response to stimuli, no statistically significant differences were found between the study groups.

## Results

### Influence of factors.

The significance of a sound stimulus for each of the factors (group, GCS, a type of a stimulus, hemisphere, area, channel) was determined, statistically significant results were obtained for GCS, group and a stimulus in the course of the HFD analysis, according to the Multivariate test data for the bands in the frequency ranges of 2–20, 2–7, 8–13, and 14–19 Hz.

Based on the results of one-way MANOVA, Table 2 shows the results of the statistical significance of Wilks’ λ. Significance was assessed on the base of the “p” column for these factors. The results of the Wilks’ λ test suggest that the HFD values are highly dependent on the group, on a stimulus, and the interaction of the group factors and stimuli (p<0.0005).

**Table 2:** Results of Wilks’ λ statistics for a factor effect in three groups tested in the frequency ranges of 2–20, 2–7, 8–13 Hz

Factor	**Wilks’** λ	F-test	df	р
Group	0.993	3.278	8	0.0005
Stimulus	0.980	3.966	20	0.0005
Group and stimulus	0.871	13.758	40	0.0005

It is important to note that alpha correction was performed to calculate multivariate ANOVA values — the Bonferroni correction was applied at each stage. Three comparisons were made in succession (group 1 was compared with group 2, group 2 with group 3, and group 1 with group 3). The permissible value was p=(1––0.951)/3=0.017. After the alpha correction, statistically significant intergroup differences were obtained for the ranges of 2–20 and 8–13 Hz. No significant differences in the “group” factor were found in the 14–19 Hz band. At the frequency of 2–7 Hz, the differences were close to significant (p=0.021). Statistically significant differences were obtained for the mean HFD values for all the 7 states (6 stimuli and the resting state) between the severe DAI group and the mild DAI and control groups; the differences between the control group and the mild DAI group were not statistically significant.

### HFD in the resting state.

The p-value for the Wilks’ λ score was found to be less than 0.0005. A statistically significant intergroup effect was achieved for HFD in the frequency range of 2–20 Hz (F(2.653)=30.655; p<0.0005; partial η2=0.086); 8–13 Hz (F(2.653)=26.999; p<0.0005; partial η2=0.076); 2–7 Hz (F(2.653)=14.568; p<0.0005; partial η2=0.043).

**Figure 1:**
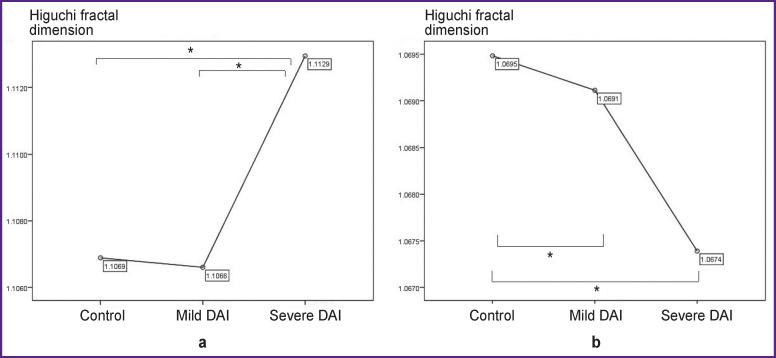
Mean value of Higuchi fractal dimension in the resting state in the frequency bands of 2–20 Hz (a), 8–13 Hz (b); * p<0.017

The highest HFD values (2–20 Hz) were observed in the group of severe DAI compared to the control group and mild DAI (2–7 Hz); no differences were found between the control group and the mild DAI group. With the positive Leuven test, the Game’s–Howell test was applied for alpha correction in the range of 2–7 Hz. The HFD values in the severe DAI group were higher than in the control and mild DAI groups; there were no differences between the control group and the mild DAI group (Figure 1 (a)). At the frequency of the alpha rhythm (8–13 Hz), the highest HFD values were obtained in the control group as compared to the mild DAI group (p<0.0005) and the severe DAI group (p<0.0005), the HFD values in the mild DAI group were more statistically significant than in the group with severe DAI (p<0.015) (Figure 1 (b)).

### HFD on presentation of sound emotional stimuli.

The response to stimuli in all the groups and at all the frequencies was manifested by higher HFD values in the left hemisphere as compared to the right one. To assess the response to sound stimuli, we subtracted the HFD value in the resting state from the HFD value for each stimulus in each of the three groups. The multivariate analysis of variance (MANOVA) was applied to the obtained data. The results of the multivariate test showed a high statistical significance for the factors: “group” (Wilks’ λ=0.856; F(6.7832)=105.15; p<0.0005), “stimulus” (Wilks’ λ=0.993; F(15.10810)=1.735; p<0.0005) and the combined factor “group and stimulus” (Wilks’ λ=0.960; F(30.11494)=5.295; p<0.0005). A statistically significant difference in the HFD values between the groups was achieved at all frequency ranges. At this stage of the study we excluded the results in the beta rhythm range from the analysis due to the influence of high tone, manifested by the presence of muscle artifacts on the electroencephalogram in the DAI groups.

### 2–20 Hz.

**Figure 2:**
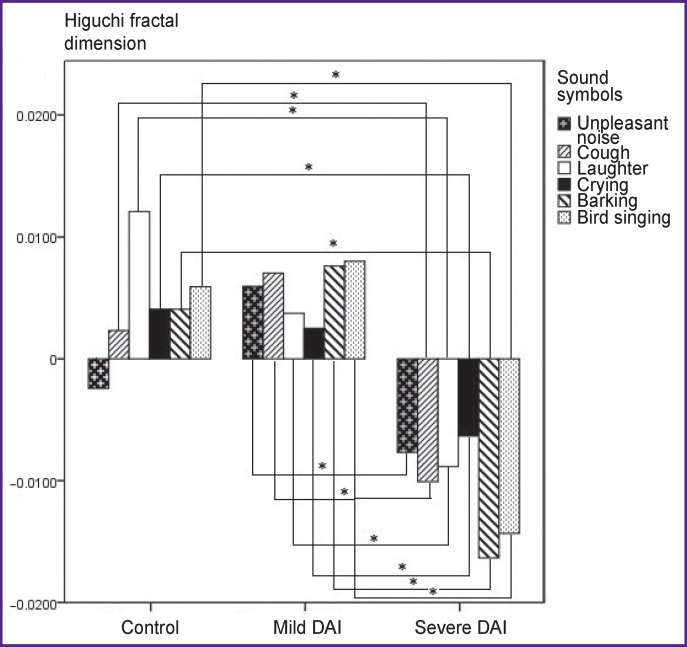
Values of Higuchi fractal dimension on presentation of stimuli relative to the resting state in the 2–20 Hz frequency band; * p<0.017

At the frequency range of 2–20 Hz, the HFD values on presentation of stimuli regarding the resting state in the control group were distributed in the positive half-plane, with the exception of the HFD value for “unpleasant noise”; in the mild DAI group, the HFD value for each of the stimuli was higher than the HFD value in the resting state; in the group with severe DAI the HFD values for each of the stimuli were significantly lower than in the resting state (Figure 2). Statistically significant differences in the HFD values between the control group and the group of severe DAI were established for the stimuli: “cough” (p=0.0005), “laughter” (p=0.0005), “crying” (p=0.001), “barking” (p=0.0005), “bird singing” (p=0.0005). There was no statistically significant difference in the HFD values between the control group and the mild DAI group. A statistically significant difference in the HFD values for stimuli between the groups with mild and severe DAI was observed for all the stimuli: high values were obtained for the stimuli “unpleasant noise”, “cough”, “laughter”, “barking”, “bird singing” — p=0.0005; for the “crying” stimulus — p=0.006.

### 8–13 Hz.

The HFD values in the control group differed with a statistical significance from the HFD values in the group of severe DAI for the stimuli: “unpleasant noise” (p=0.002), “cough” (p=0.0005), “laughter” (p=0.0005), “crying” (p=0.0005), “barking” (p=0.0005), “bird singing” (p=0.0005).

**Figure 3:**
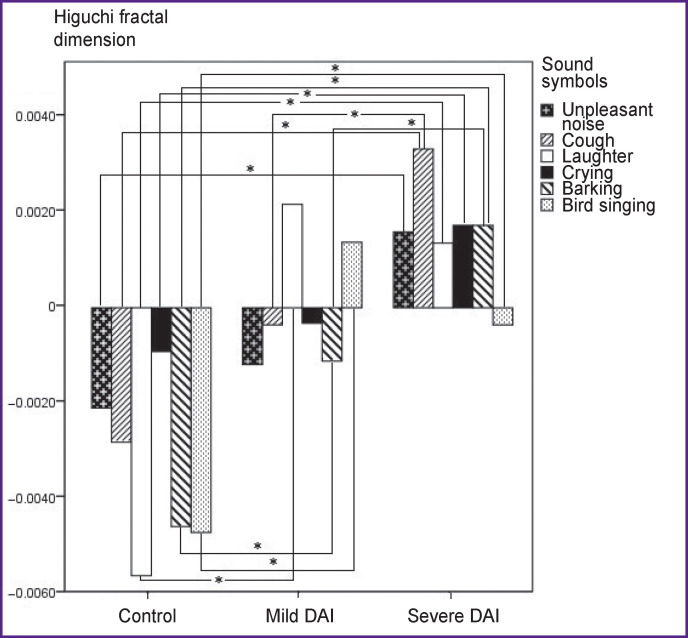
Values of Higuchi fractal dimension on presentation of stimuli relative to the resting state in the 8–13 Hz frequency band; * p<0.017

Significant differences between the control group and the mild DAI group were established for the stimuli “laughter” (p=0.0005), “barking” (p=0.001), “bird singing” (p=0.0005). Statistically significant differences in the HFD values between the groups of severe and mild DAI were observed for the stimuli “cough” (p=0.001) and “barking” (p=0.005) (Figure 3).

### 2–7 Hz.

**Figure 4:**
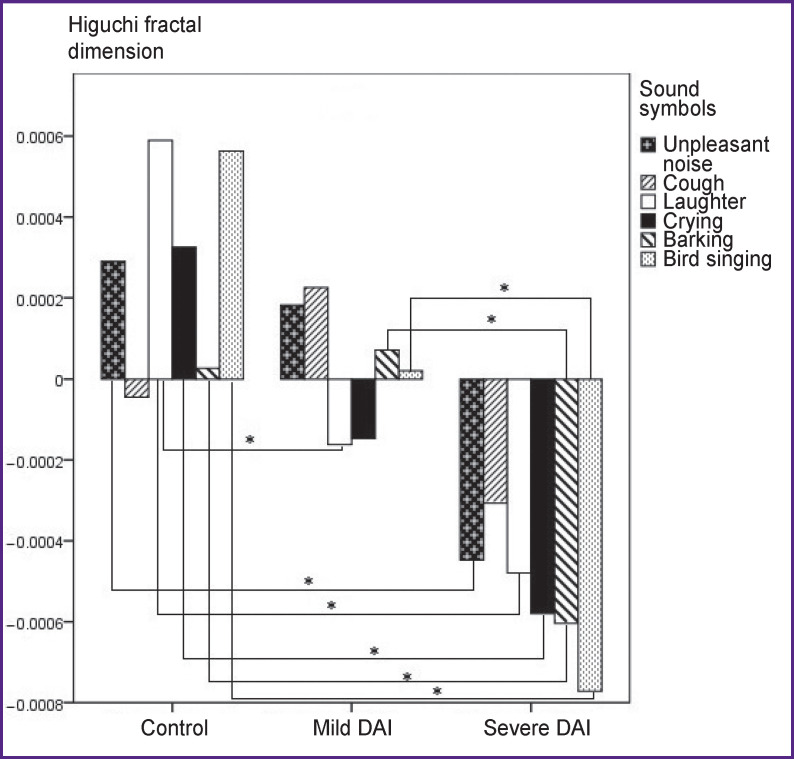
Values of Higuchi fractal dimension on presentation of stimuli relative to the resting state in the 2–7 Hz frequency band; * p<0.017

The distribution of the difference in the HFD values for sound stimuli relative to the resting state is shown in Figure 4. No statistically significant differences in the HFD values for the “cough” sound were observed between the groups.

The highest HFD values were observed for the sound “laughter” in the control group and differed from the mild DAI group (p=0.003) and the severe DAI group (p=0.0005). The HFD values between the control group and the severe DAI group differed for all the stimuli: “unpleasant noise” (p=0.008), “laughter” (p=0.0005), “crying” (p=0.0005), “barking” (p=0.014), “bird singing” (p=0.0005), except for the “cough” stimulus. Statistically significant differences between the control group and the mild DAI were obtained for the “laughter” stimulus (p=0.003). The HFD values between the severe DAI and mild DAI groups differed for the “barking” (p=0.007) and “bird singing” (p=0.001) stimuli.

## Discussion

The Higuchi’s method is one of the most reliable methods for assessing the complexity of long EEG segments; it has proved itself to be a method for differentiating the level of consciousness [20–22]. The use of sound stimuli in our work was intended to provoke positive and negative emotions in the subjects. The response to stimuli in all the three groups had higher indicators of signal complexity in the left hemisphere in comparison with the right one. An increase in the complexity of the EEG signal in response to emotionally significant stimuli in healthy subjects was found to correspond to the theta rhythm, while in the group with severe DAI, a similar increase in the complexity of responses was observed at the frequency of the alpha rhythm. The HFD values in the mild DAI group differed from the results in the control group and the severe DAI group and had intermediate values.

The study by Ruiz-Padia and Ibáñez-Molina [22] suggested that the highest HFD values were obtained in response to humorous videos at a frequency of 0.3–40 Hz. In our study, the highest HFD values on a laughter stimulus were obtained in the control group in the range of 2–20 Hz. Higher HFD values in the work of the Spanish colleagues [22] are associated with a complicated visual-sound effect of a stimulus in comparison with a simpler effect of only a sound stimulus. Difficulties in assessing the emotion perception in unconscious patients determined the choice of stimulus material in our study and the use of the HFD analysis in functional states provoked by sound stimuli, be it laughter, crying, or an unpleasant sound. Due to these subjectively independent features, the complexity of the EEG signal was found not only to differ between the studied groups, but also to change depending on a frequency range. The further study will be aimed at increasing the number of subjects and searching for subject-independent EEG patterns to remove the effect of high individual variability. Studies of cognitive response in unconscious patients over time will help to create more advanced diagnostic methods for assessing higher psychic functions.

## Conclusion

Active information processing including that in response to long-term sound stimuli changes the fractal dimensionality of the EEG signal increasing its complexity. The Higuchi’s method makes it possible to determine and assess this value. The Higuchi fractal dimension can be used as an additional diagnostic tool to define syndromes associated with the perception of emotionally significant auditory information in patients with brain damage.
